# The Relation of Public Parks to Public Health

**Published:** 1899-06

**Authors:** Orlando B. Douglas

**Affiliations:** New York, N. Y.; Fellow New York Academy of Medicine


### Abstracts.

### THE RELATION OF PUBLIC PARKS TO PUBLIC HEALTH.

By ORLANDO B. DOUGLAS, New York, N. Y.
Fellow New York Academy of Medicine.

HAVE public parks an effect on public health? Has the money expended for them brought adequate return to the people in the way of lengthened life or better health? Figures are wanting—or are inadequate—to prove that parks give health, or to show the number of years added to human life, and the amount and degree of health given to men, women and children by their influence. Tables may show who and how many people visit the parks, and how many hours are spent there. But statistics fail to include—and the scales are yet to be made that can weigh—the influence and sunshine of pure air, the effect of balsamic emanations, of ozone from the pines, odors of flowers and grasses, that are made accessible to the people by public parks. Who can tell from what ills he has been saved by the rest and quiet obtained in nature's sanatorium; by her soothing lullaby sung in the treetops!

Gold that brings health can never be ill spent.

The subject of public sanitation concerns every man, woman and child; touches every hearth and home; involves every institution, its power, production and perpetuity; every trade and occupation; domestic purity and happiness; the duties and privileges of property; national health, morals and longevity; the exercise of charity and the growth and fruitage of our common Christianity. Its end is to preserve and improve man physically, and thus purify and perfect the immortal. It is immediate in its action and far-reaching in effect. The great qualities of any people depend, in large measure, upon the health and physique of individuals.

Last November Warren Manning sent out a large number of letters to leading physicians in this country, and a few in foreign countries, asking for an answer to the following questions, viz. : First, has the enlargement and improvement of the public park system in cities resulted in diminishing the rate of increase in nervous diseases, or in the improvement of the general health in cities? Second, Is it true that quiet, pastoral scenes, and the absence of noise and excitement, have a beneficial effect upon persons afflicted with nervous diseases? Third, do you find that brilliant color-effects—such as are often provided in flower gardens—are a source of irritation to persons thus afflicted?

Dr. Raffegean, Vesinet, France, who has given much attention to the effects of colors upon nervous and insane people, and reports many interesting cases in illustration, says: "I am convinced that the enlargement and development of public parks has tended to diminish the progress of nervous diseases, and to improve the health of the inhabitants of cities, but I know of no statistics regarding this. It is undeniable that their removal from noises and excitement has a beneficial effect on those who are victims of their nerves. The bright colors which irritate nervous people are the red, and the colors that approach this, *i. e.*, orange and yellow; while violet and the other colors of the rainbow are calming. For this reason, nature has given to the leaves of trees and plants the color of green, while the sky is blue and the sea is azure. Nothing rests the eyes more than a beautiful field."

The two Buffalo physicians who responded sent in the following replies:

Dr. A. W. Hurd, Buffalo, N. Y., says: "Anything which promotes the general physical health of patients is an advantage in the cure of mental diseases. For that reason parks, gardens, flowers, farms, with the accompanying fresh air, oxygen, exercise, are of advantage. It is hard to give any statistics as to how much more of nervous diseases would prevail if we had no parks, but you may safely rest on this general principle."

Dr. Wm. C. Krauss, Buffalo, N. Y., believes the park system materially lessens the functional nervous cases and their severity, but cannot see how they can influence the chronic or organic diseases of the nervous system. That quiet, pastoral scenes exert a beneficial influence on functional nervous diseases goes without saying: we send such cases to the country for rest and quiet.—*Transactions American Park and Outdoor Art Association, 1898.*

